# Knockdown of the Sodium-Dependent Phosphate Co-Transporter 2b (NPT2b) Suppresses Lung Tumorigenesis

**DOI:** 10.1371/journal.pone.0077121

**Published:** 2013-10-23

**Authors:** Seong-Ho Hong, Arash Minai-Tehrani, Seung-Hee Chang, Hu-Lin Jiang, Somin Lee, Ah-Young Lee, Hwi Won Seo, Chanhee Chae, George R. Beck, Myung-Haing Cho

**Affiliations:** 1 Laboratory of Toxicology, College of Veterinary Medicine, Seoul National University, Seoul, Korea; 2 School of Pharmacy, China Pharmaceutical University, Nanjing, China; 3 Laboratrory of Pathology, College of Veterinary Medicine, Seoul National University, Seoul, Korea; 4 Division of Endocrinology, Metabolism and Lipids, Emory University School of Medicine, Atlanta, Georgia, United States of America; 5 Graduate School of Convergence Science and Technology, Seoul National University, Suwon, Korea; 6 Graduate Group of Tumor Biology, Seoul National University, Seoul, Korea; 7 Advanced Institute of Convergence Technology, Seoul National University, Suwon, Korea; Cincinnati Children’s Hospital Medical Center, United States of America

## Abstract

The sodium-dependent phosphate co-transporter 2b (NPT2b) plays an important role in maintaining phosphate homeostasis. In previous studies, we have shown that high dietary inorganic phosphate (Pi) consumption in mice stimulated lung tumorigenesis and increased NPT2b expression. NPT2b has also been found to be highly expressed in human lung cancer tissues. The association of high expression of NPT2b in the lung with poor prognosis in oncogenic lung diseases prompted us to test whether knockdown of NPT2b may regulate lung cancer growth. To address this issue, aerosols that contained small interfering RNA (siRNA) directed against NPT2b (siNPT2b) were delivered into the lungs of K-*ras* ^LA1^ mice, which constitute a murine model reflecting human lung cancer. Our results clearly showed that repeated aerosol delivery of siNPT2b successfully suppressed lung cancer growth and decreased cancer cell proliferation and angiogenesis, while facilitating apoptosis. These results strongly suggest that NPT2b plays a role lung tumorigenesis and represents a novel target for lung cancer therapy.

## Introduction

Lung cancer is the leading cause of cancer death worldwide. Despite recent developments in lung cancer therapy, lung cancer remains associated with severe morbidity and the five-year survival rate has improved only minimally over time [1]–[4]. Therefore, it is essential to identify novel target molecules for the development of new lung cancer therapies.

Among the three sodium-dependent phosphate co-transporters (NPT1, NPT2, and NPT3), NPT2 and NPT3 have been identified as transporters of inorganic phosphate (Pi) in mammalian cells. NPT2 isoforms include NPT2a, NPT2b, and NPT2c. NPT2b is a tissue-specific transporter that is highly expressed in the lungs [5]–[9], and plays crucial roles in maintaining overall phosphate homeostasis, which is important for proper cellular functioning and signal transduction [10]–[12].

Recent reports have described NPT2b as a potential molecular marker for various types of cancer. Hashimoto *et al.* [13] indicated that NPT2b may be a promising marker in the analysis of the histopathogenesis of lung cancer. NPT2b overexpression has been reported in papillary thyroid cancer and breast cancer [14]–[16]. We have also previously shown that high dietary phosphate increases NPT2b expression and stimulates lung tumorigenesis as well as altering Akt-ERK signaling, protein translation, and angiogenesis [17]–[20]. This raised the question of whether regulation of cellular phosphate transport in the lungs by knockdown of NPT2b would have beneficial therapeutic effects in lung cancer. To address this question, we used a small interfering RNA (siRNA) to silence the expression of NPT2b (siNPT2b), and tested the consequences on tumor growth in K-*ras* ^LA1^ model mice. These mice carry a latent and activated K-*ras* allele (G12D) which results in development of lung adenocarcinoma [21], [22].

## Materials and Methods

### *In vivo* Aerosol Delivery

All the animal experiments were approved by the care and use of laboratory animals of Seoul National University (SNU-111006-2). K-*ras* ^LA1^ mice were obtained from the National Cancer Institute-mouse repository (Frederick, MD, USA). The mice were housed under a standard light/dark cycle at 23°C±2°C with a relative humidity of 50%±10%. In this study, 10-week-old female K*-ras* ^LA1^ mice were used (six mice per group). All mice were fed the same standard diet containing normal Pi levels (0.5% Pi). The mice were divided into three groups: the control (Con) group was untreated, and the other two groups were exposed to aerosols that contained either GPT-SPE/siNPT2b (siNPT2b group) [5′-UACAGAAUACUUCAUAACUUA-3′] or GPT-SPE/Scr (Scr group) [5′- GAUAGCAAUGACGAAUGCGUATT-3′]. The poly (amino ester) (PAE) that was used as the DNA carrier and was based on glycerol propoxylate triacrylate (GPT) and spermine (SPE) complexes was prepared as described previously [23]. The mice were placed in a nose-only exposure chamber (Dusturbo, Seoul, Korea) and exposed to aerosol that was generated using a patented nebulizer (Korean patent #20304964) containing GPT-SPE/siRNA complex solution (containing 0.5 mg of siRNA), twice a week for four weeks. At the end of the study, the 14-week-old K-ras^LA1^ mice were anesthetized with 15 mg/kg of Zoletil (Laboratoires Virbac, Carros, France) and 3 mg/kg of xylazine (Laboratoires Calier, Barcelona, Spain), and their lungs were collected. During the autopsy procedure, the neoplastic lesions on the entire lung surface were counted, and the lesion diameter was measured using digital calipers.

### TUNEL Assay

Lung tissue slides were deparaffinized in xylene and rehydrated through an alcohol gradient. After washing, nicked DNA ends were labeled via the terminal TUNEL method using an *in situ* Cell Death Detection kit (Roche, Basel, Switzerland), following the manufacturer’s instructions. The tissue sections were counterstained with methyl green (Trevigen, Gaithersburg, MD, USA).

### Western Blot Analysis

Total right cranial lung lobes of mice, including the tumors, were homogenized for Western blot analysis. The total protein concentration in the homogenized lung samples was determined using the Bio-Rad Protein Assay reagent (Bio-Rad, Hercules, CA, USA). Western blotting was performed following a previously described procedure [23]. Anti-NPT2b (N0035-26C) antibody was purchased from US Biological (Salem, MA, USA). Antibodies against NPT2a (V-20), NPT2c (L-15), VEGF (C-1), PCNA (PC-10) and actin (I-19) were purchased from Santa Cruz Biotechnology (Santa Cruz, CA, USA). Bands were detected using the LAS-3000 luminescent image analyzer (Fujifilm, Tokyo, Japan) and quantified using Multi Gauge version 2.02 software (Fujifilm, Tokyo, Japan).

### Histopathology and Immunohistochemistry (IHC)

Because experiments and analyses needed to be performed on non-damaged lung tissues, non-perfused fixed lungs were embedded in paraffin, and the paraffin-embedded tissue sections were cut and transferred to plus slides. For histological analysis, the tissue sections were stained with hematoxylin and eosin (H&E) and were then examined by a light microscopy. For immunohistochemistry IHC, the tissue sections were deparaffinized in xylene and rehydrated through alcohol gradients. Then the sections were washed and incubated in 3% hydrogen peroxide (AppliChem, Boca Raton, FL, USA) for 10 min to quench endogenous peroxidase activity. After washing in phosphate-buffered saline (PBS), sections were incubated with 3% bovine serum albumin (BSA) in PBS for 1 h at room temperature to block non-specific binding sites. Primary antibodies were applied to the tissue sections and incubated overnight at 4°C. The following day, the tissue sections were washed and incubated with secondary horseradish peroxidase (HRP)-conjugated antibodies for 3 h at room temperature. After washing, sections were counterstained with Mayer’s hematoxylin (DAKO, Carpinteria, CA, USA) and washed with xylene. The cover slips were mounted using permount, and the slides were imaged under a light microscope (Carl Zeiss, Thornwood, NY, USA).

### Real-time Quantitative PCR

Total accessory lung lobes of mice including tumors were homogenized for RNA analysis. Human lung tissues were obtained from KLTB (Korea Lung Tissue Bank, Seoul, Korea) and all of the procedures using human samples were approved by Instutional Review Board of Seoul National University (SNUIRB-E1201/001-001) as well as KLTB (KU Guro Gene Bank 2012-004). Total RNA was isolated using the QuickGene RNA kit (Fujifilm’s Life Science System, Tokyo, Japan) and then converted to cDNA by SuPrimeScript RT Premix (GeNet Bio, Cheonan, Korea). Real-time quantitative (qPCR) was carried out with the CFX96^T^ Real-time System (Bio-Rad, Hercules, CA, USA). Each cDNA was amplified with a specific primer and Prime Q-Mastermix (GeNet Bio, Choenan, Korea) under the following cycling conditions: initial denaturation at 94°C for 10 min, ; 40 cycles, each consisting of denaturation at 94°C for 30 s, annealing at 59°C for 30 s, extension at 72°C for 45 s; and final extension at 72°C for 5 min. The products were analyzed with the Bio-Rad CFX Manager Version 2.1 software.

The sequences of the primers that were used for the qPCR were as follows: human NPT2b, forward (5′-CAAGGAGAACATCGCCAA-3′) and reverse (5′-GACCAGCAGGGAGAGTAT-3′); human GAPDH, forward (5′-GCCCAATACGACCAAATC-3′) and reverse (5′-ACTCAGCCGCATCTT-3′); mouse NPT2b, forward (5′-GAACCTCCATCACCAACA-3′) and reverse (5′-AGAGCACGAACACAGAGA-3′); mouse NPT2a, forward (5′-CTTCAACATCCGAGGTGG-3′) and reverse (5′-ATCCGAATGAGACTGTGA-3′); mouse NPT2c, forward (5′-GCCATTGTCTACCTACTATTAACC-3′) and reverse (5′-ACATTAACCAGGATGATAAGGAG-3′); and mouse β-actin, forward (5′-TTTCCAGCCTTCCTTCTTGGGTATG-3′) and reverse (5′-CACTGTGTTGGCATAGAGGTCTTTAC-3′).

### Statistical Analyses

Statistical analyses were performed using Student’s *t*-test for experiments that consisted of two groups (Graphpad Software, San Diego, CA, USA) and data are presented as the mean ± SEM. *P-values<0.05 were considered significant, **P<0.01 and ***P<0.001 were considered highly significant compared with the corresponding control values.

## Results and Discussion

Current progress in synthetic siRNA delivery to specific cell types *in vivo* strongly supports the therapeutic potential of RNAi-based methods for cancer treatment [23]–[26]. Several methods have been developed to deliver siRNA to the lungs. The aerosol delivery system represents a non-invasive method with the potential to effectively deliver siRNA to the lungs. However, because of the size, low stability, and rapid excretion, efficient delivery of siRNA requires a vector [27], [28]. A number of non-viral vectors have recently been reported for the introduction of oligomers or nucleotides into various tissues [29], [30]. Among these, we have previously shown that GPT-SPE is an effective delivery vehicle for siRNA *in vitro* and *in vivo* [23].

**Figure 1:**
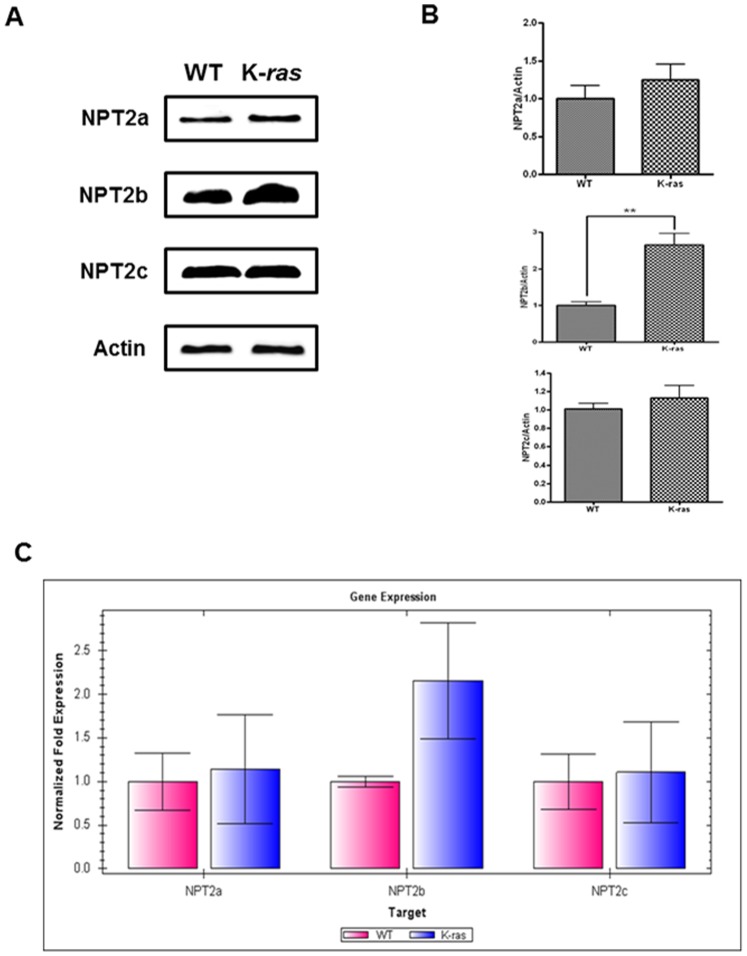
Expression levels of the NPT2 transporters in the lungs of WT and K*-ras* ^LA1^ mice. All wild-type mice (WT) and K-ras^LA1^ mice were 10 week old female. (**A**) Western blot analysis of NPT2a, NPT2b, and NPT2c in the lungs of the WT and K*-ras* ^LA1^ mice. (**B**) The bands-of-interest were further analyzed via densitometry. Each bar represents the mean±SEM (*n* = 3). **P<0.01 relative to corresponding control values. (**C**) Quantitative real-time PCR analysis of NPT2a, NPT2b, and NPT2c in the lungs of the WT and K*-ras* ^LA1^ mice. Each bar represents the mean±SEM (*n* = 4). (WT = wild-type normal mice and K-*ras = *K*-ras* ^LA1^ mice).

We first examined, the expression levels of three different transporters (the NPT2 family) in the lungs of 10-week-old K-*ras* ^LA1^ and wild-type (WT) mice. The results revealed increased expression of NPT2b in the lungs of K-*ras* ^LA1^ mice compared to that in the lungs of WT mice, as determined via Western blotting, densitometric analysis and qPCR (Figure 1) although the results did not reach statistical significance (Figure 1C).

**Figure 2:**
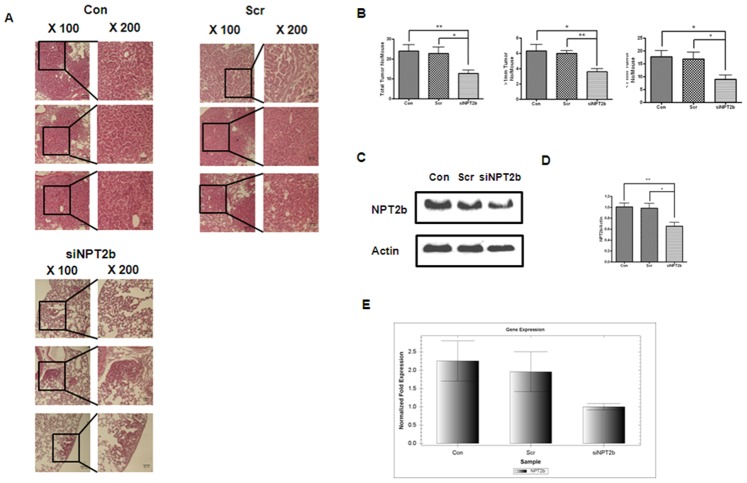
Tumor histopathology and expression level of NPT2b in the lungs of siNPT2b-delivered mice. (**A**) Histological examination of the lungs of the 10-week-old K*-ras* ^LA1^ mice (magnification×100 and ×200; the bars represent 100 µm and 50 µm each). (**B**) Tumor numbers in the lungs of the six K*-ras* ^LA1^ mice. Each bar represents the mean±SEM (*n* = 6). (**C**) Western blot analysis of NPT2b in the lungs of the K*-ras* ^LA1^ mice after the aerosol delivery. (**D**) Densitometric analysis was performed on the Western blot bands in five separate mice. Each bar represents the mean±SEM (*n* = 5). *P<0.05, **P<0.01 relative to Con or Scr. (**E**) Quantitative real-time PCR analysis of NPT2b in the lungs of the K*-ras* ^LA1^ mice. Each bar represents the mean±SEM (*n* = 6). (Con = control; Scr = scrambled control; and siNPT2b = siNPT2b-delivered group).

The association of a high NPT2b expression with poor prognosis in oncogenic lung diseases and the potential importance of Pi prompted us to study the possible inhibitory effect of down-regulated NPT2b on tumor growth. Hence, siRNA targeting NPT2b was delivered into the lungs of K-*ras* ^LA1^ mice through inhalation twice a week for four weeks. The repeated aerosol delivery of siNPT2b significantly decreased the number and size of the lung surface tumor lesions (Figures 2A and 2B). The nodules that developed in the lungs of the control (Con) and scrambled control (Scr) groups were generally alveolar/bronchial adenocarcinoma and adenoma and their numbers were greater compared to those for the siNPT2b group (Table 1). Most of the nodules in the lungs of the siNPT2b group were adenomas and hyperplastic lesions and these were significantly lower in the siNPT2b mice relative to either Con or Scr siRNA groups (Table 1). Moreover, aerosol-delivered siNPT2b decreased NPT2b expression levels, as detected via Western blotting, densitometric analysis and qPCR (Figures 2C–2E). Although the decrease did not reach statistical significance the functional relevance is demonstrated by the decrease in tumor burden (Table 1).

**Table 1:** Summary of tumor incidence in the lungs of K-*ras* ^LA1^ mice.

Group	Mice identification	No. of Adenocarcinoma	No. of Adenoma	No. of hyperplasia foci
Con	1	–	3	6
	2	2	1	9
	3	–	3	7
	4	–	2	5
	5	1	2	10
	6	1	1	3
Avg		0.67	2.00	6.67
STD		0.82	0.89	2.58
Scr	1	–	2	3
	2	–	2	5
	3	2	3	8
	4	–	1	7
	5	1	2	9
	6	–	4	6
Avg		0.50	2.33	6.33
STD		0.84	1.03	2.16
siNPT2b	1	–	1	2
	2	–	–	2
	3	–	1	4
	4	–	–	3
	5	–	2	2
	6	–	–	3
Avg		0	0.67*	2.67**
STD		0	0.82	0.82

* *P*<0.05,

** *P*<0.01 relative to both Control (Con) and scrambled control (Scr);

siNPT2b = siNPT2b-delivered group.

Ten-week-old female K*-ras* ^LA1^ mice were exposed to aerosols containing siNPT2b, twice a week for four weeks. At the end of the test period, the K*-ras* ^LA1^ mice were sacrified, the lungs were collected, and tumor incidence investigated.

**Figure 3:**
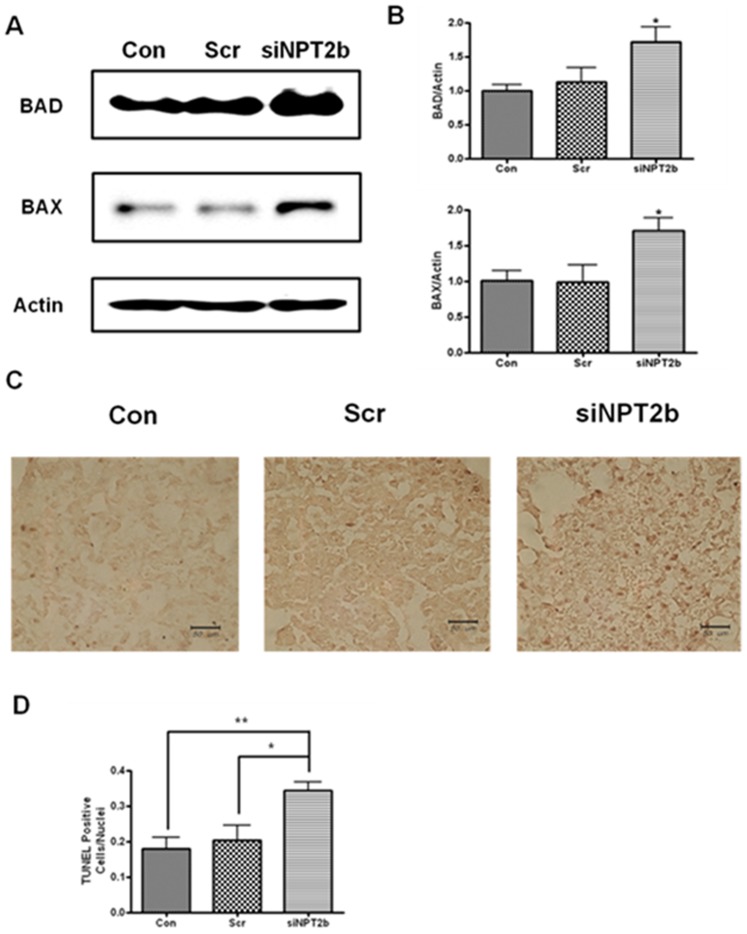
Effects of the aerosol-delivered siNPT2b on the apoptotic proteins. (**A**) Western blot analysis of the BAX and BAD proteins. (**B**) Densitometric analysis of the Western blots. (**C**) TUNEL assay on the tumor lesions of lungs of the K*-ras* ^LA1^ mice. (**D**) Graph representing the number of TUNEL-positive cells. Each bar represents the mean±SEM (*n* = 4). *P<0.05, **P<0.01 relative to Con or Scr. (Con = control; Scr = scrambled control; and siNPT2b = siNPT2b-delivered group).

The development of tumors depends decreased apoptosis of cancer cells. Therefore, increasing apoptosis is a promising strategy for suppressing tumor progression [31]–[32]. To assess whether inhibition of lung cancer growth by knockdown of NPT2b is correlated with the induction of apoptosis, the abundance of apoptosis-related proteins was analyzed. The results indicated that aerosol-delivered siNPT2b significantly increased BAX and BAD protein levels, as determined via Western blotting and densitometric analysis (Figures 3A and 3B). TUNEL assay data showed a higher number of TUNEL-positive cells in the lungs of the siNPT2b group and the quantitative and statistical analyses confirmed this result (Figures 3C and 3D). These data demonstrate that siNPT2b can facilitate apoptosis in the lungs of K-*ras* ^LA1^ mice.

**Figure 4:**
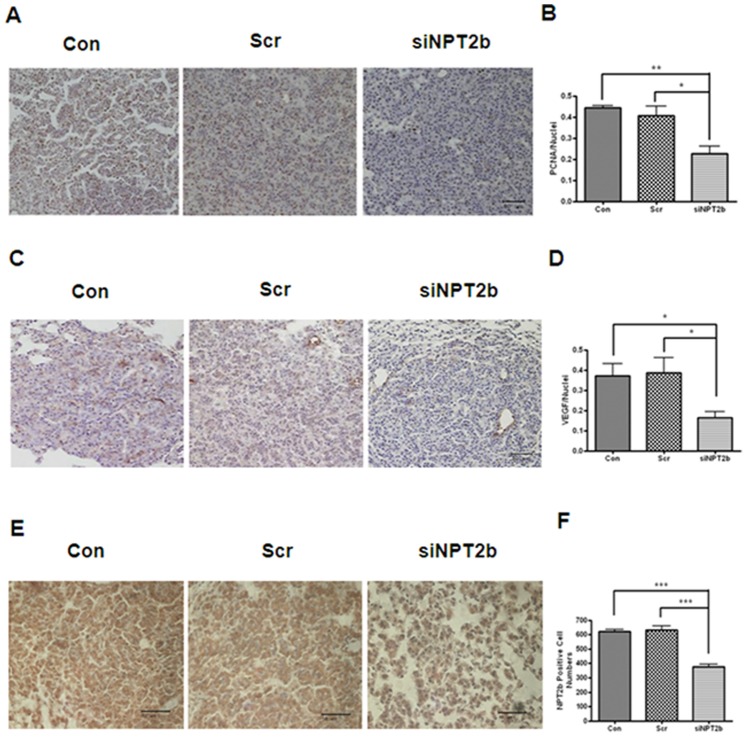
Effects of the aerosol-delivered siNPT2b on tumor cell proliferation and angiogenesis. (**A**) Immunohistochemistry analysis of PCNA in the lungs (magnification×200; *bar* = 50 µm). (**B**) Graph representing the number of PCNA-positive cells. Each bar represents the mean±SEM (*n* = 4). (**C**) Immunohistochemistry analysis of VEGF (magnification×200; *bar* = 50 µm). (**D**) Graph representing the number of VEGF-positive cells. Each bar represents the mean±SEM (*n* = 4). (**E**) Immunohistochemistry analysis of NPT2b (magnification×400; *bar* = 50 µm). *P<0.05 was considered more significant than the corresponding control values. (**F**) Graph representing the number of NPT-2b-positive cells. Each bar represents the mean±SEM (*n* = 4). *P<0.05, **P<0.01 and ***P<0.001 relative to Con or Scr. (Con = control; Scr = scrambled control; and siNPT2b = siNPT2b-delivered group).

Uncontrolled proliferation and angiogenesis are two of the major characteristics of tumorigenesis [32]. To determine the effect of siNPT2b on tumor cell proliferation, the expression level of PCNA was analyzed. IHC analysis clearly showed that aerosol delivery of siNPT2b decreased PCNA expression levels (Figure 4A). Angiogenesis is required for invasive tumor growth and plays important roles in cancer progression [33]. Therefore we also investigated the expression level of angiogenesis-related protein in the lung. The results showed that siNPT2b decreased VEGF levels, as detected via IHC analysis (Figure 4C). The expression level of NPT2b also decreased in the siNPT2b-delivered group (Figure 4E). Quantitation of PCNA, VEGF and NPT2b by IHC clearly confirmed the decreased expression of these proteins in the lungs of the siNPT2b-treated group (Figures 4B, 4D and 4F).

These results clearly showed that siNPT2b delivery decreased cell proliferation and angiogenesis in the lung tissue. In addition, we found that NPT2b expression is elevated in human lung cancer tumor samples (stages I and III adenocarcinoma), compared to the levels in normal lung tissues,; however, NPT2b expression is only slightly higher in lung tissues with stage II adenocarcinoma than in normal lung tissues, as determined via qPCR (Figure S1). Lederer *et al.* [34] indicated that the NPT2b protein is expressed in lung cancer and our studies found increased expression of NPT2b in human lung adenocarcinoma and that its expression correlates with certain tumor stages.

These results further support our hypothesis that NPT2b is an important therapeutic target and suggest that NPT2b inhibition by using siRNA could be a powerful lung cancer treatment strategy. This study showed that the aerosol delivery system can be used to deliver synthetic siRNA and offers an innovate approach to deliver siRNA as an effective cancer therapy. We also demonstrated that aerosol delivery of siRNA against NPT2b successfully suppressed lung cancer growth, suggesting that NPT2b-knockdown and thus, regulation of phosphate consumption, may be a promising form of treatment for lung cancer.

## Supporting Information

Figure S1 **Expression levels of the NPT2b transporter in human lungs.** Quantitative real-time PCR analysis of NPT2b in human normal and adenocarcinoma lung tissues. Each bar represents the mean±SEM (n = 4) (Normal = normal lung tissues; I = lung cancer tumor samples (stage I adenocarcinoma); II = lung cancer tumor samples (stage II adenocarcinoma); and III = lung cancer tumor samples (stage III adenocarcinoma).
(TIF)
