# Correction: Knockdown of the Sodium-Dependent Phosphate Co-Transporter 2b (NPT2b) Suppresses Lung Tumorigenesis

**DOI:** 10.1371/annotation/08286cd8-527f-4f14-856f-57267107efa8

**Published:** 2014-01-09

**Authors:** Seong-Ho Hong, Arash Minai-Tehrani, Seung-Hee Chang, Hu-Lin Jiang, Somin Lee, Ah-Young Lee, Hwi Won Seo, Chanhee Chae, George R. Beck, Myung-Haing Cho

The sixth author's name was written incorrectly. It should be Ah Young Lee.

The listed affiliation for author George R. Beck, Jr. is incorrect. His correct institutional affiliation is: Emory University, Department of Medicine, Division of Endocrinology, Atlanta, Georgia, 30322, USA.

The funding statement contains two errors. The NRF grant (2012M3A9B6055304) was funded by the Ministry of Science, ICT & Future Planning of Korea (previously the Ministry of Education, Science, and Technology of Korea). Author George Beck was also supported by a grant from the National Cancer Institute/NIH (CA136716). The updated funding statement reads: 

"This research was supported by the National Research Foundation (2012M3A9B6055304), funded by the Ministry of Science, ICT & Future Planning of Korea. MHC also acknowledges the support of the Veterinary Research Institute of Seoul National University in Korea. The authors further thank the Korea Lung Tissue Bank (KLTB) of the Infrastructure Project for Basic Science of the Ministry of Science, ICT and Future Planning for providing the human lung tissues. GRB is supported by a grant from the National Cancer Institute/NIH (CA136716). The funders had no role in study design, data collection and analysis, decision to publish, or preparation of the manuscript."

Updated versions of Figures 2 and 4 with enlarged text have been included below.

Figure 2: 

**Figure:**
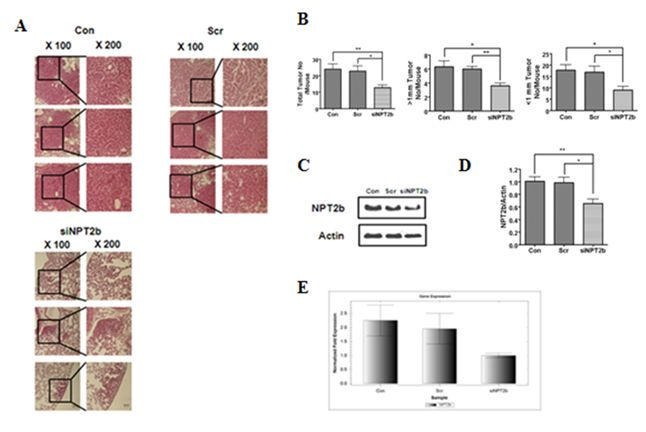


Figure 4: 

**Figure:**
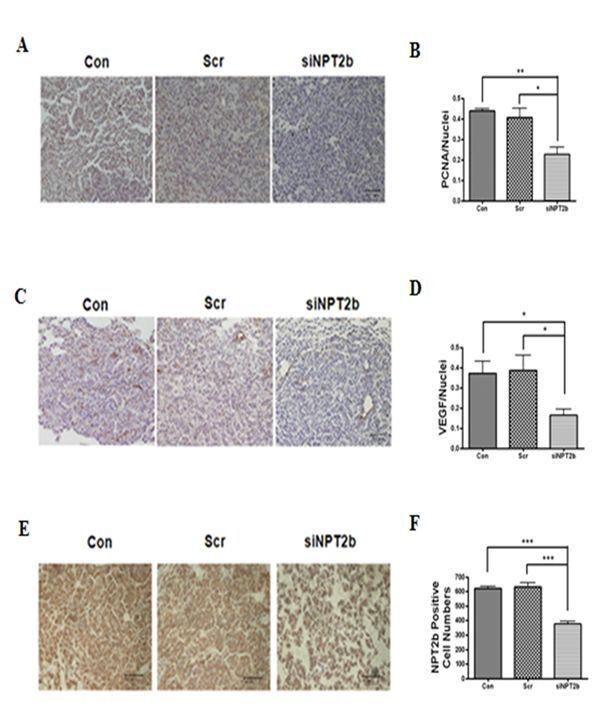


There is an error in Reference 23. The correct reference is: Jiang HL, Hong SH, Kim YK, Islam MA, Kim HJ, et al. (2011) Aerosol delivery of spermine-based poly(amino ester)/Akt1 shRNA complexes for lung cancer gene therapy. Int J Pharm. 420:256-265.

