# Supplementary figures and images for: Knockdown of the Sodium-Dependent Phosphate Co-Transporter 2b (NPT2b) Suppresses Lung Tumorigenesis
Source: PLoS One. 2013 Oct 23;8(10):e77121. doi: 10.1371/journal.pone.0077121 (PMC3806752; doi:10.1371/journal.pone.0077121)

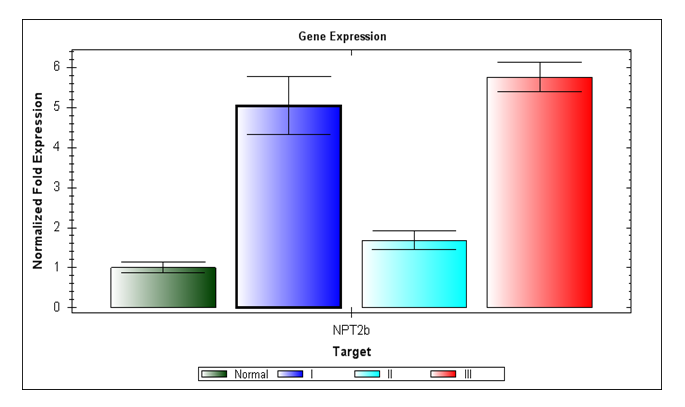

Supplement: Figure S1 — Expression levels of the NPT2b transporter in human lungs. Quantitative real-time PCR analysis of NPT2b in human normal and adenocarcinoma lung tissues. Each bar represents the mean±SEM (n = 4) (Normal = normal lung tissues; I = lung cancer tumor samples (stage I adenocarcinoma); II = lung cancer tumor samples (stage II adenocarcinoma); and III = lung cancer tumor samples (stage III adenocarcinoma). (TIF) [file pone.0077121.s001.tif]
